# Hepatitis C: Standard of Treatment and What to Do for Global Elimination

**DOI:** 10.3390/v14030505

**Published:** 2022-02-28

**Authors:** Lorenza Di Marco, Claudia La Mantia, Vito Di Marco

**Affiliations:** 1 Gastroenterology Unit, Department of Medical Specialties, University of Modena & Reggio Emilia, 41100 Modena, Italy; lor.dimarco@gmail.com; 2 Clinical and Experimental Medicine PhD Program, University of Modena & Reggio Emilia, 41100 Modena, Italy; 3 Section of Gastroenterology and Hepatology, Department of Health Promotion, Mother and Child Care, Internal Medicine and Medical Specialties (PROMISE), University of Palermo, 90127 Palermo, Italy; claudialamantia1990@gmail.com

**Keywords:** HCV infection, global elimination, HCV screening, linkage to care, direct acting antiviral drugs, sustained virological response

## Abstract

Hepatitis C virus infection has a substantial effect on morbidity and mortality worldwide because it is a cause of cirrhosis, hepatocellular carcinoma, liver transplantation, and liver-related death. Direct acting antiviral drugs available today have high efficacy and excellent safety and can be used in all patients with clinically evident chronic liver disease and in groups that demonstrate risk behaviors to reduce the spread of infection. The Global Health Strategy of WHO to eliminate hepatitis infection by 2030 assumes “a 90% reduction in new cases of chronic hepatitis C, a 65% reduction in hepatitis C deaths, and treatment of 80% of eligible people with HCV infections”. In this review effective models and strategies for achieving the global elimination of HCV infection are analyzed. The screening strategies must be simple and equally effective in high-risk groups and in the general population; fast and effective models for appropriate diagnosis of liver disease are needed; strategies for direct acting antiviral drug selection must be cost-effective; linkage to care models in populations at risk and in marginalized social classes must be specifically designed and applied; strategies for obtaining an effective vaccine against HCV infection have yet to be developed.

## 1. Introduction

Hepatitis C virus (HCV) infection influences morbidity and mortality worldwide because it is a cause of liver fibrosis, hepatocellular carcinoma (HCC), and death, and affects mortality from cardiovascular diseases [1,2,3]. In recent years, HCV infection was the cause of more than 400,000 deaths and most of these deaths are correlated with the development of liver disease complications such as cirrhosis and HCC [4,5].

An epidemiological model built in 2015 on data from 100 countries estimated a global prevalence of viremic HCV of 1%, corresponding to 71 million infected people worldwide. The highest prevalence was estimated in the Eastern Mediterranean Region (2.3%) and Europe (1.5%), while in the Western Pacific Region and Americas the estimated prevalence is less than 1%. In some geographical areas of Asia (Mongolia, Uzbekistan, and Georgia), the prevalence higher than 4%, while some African countries (Egypt and Gabon) have a prevalence greater than 6% [6].

In 2016, the World Health Organization (WHO) approved the Global Strategy to eliminate HCV infection by 2030. To achieve this target, the WHO plans to obtain “a 90% reduction in new cases of chronic hepatitis C, a 65% reduction in hepatitis C deaths, and treatment of 80% of eligible people with chronic hepatitis C infections” [7,8].

There are four steps that require specific activities and deliberate interventions to achieve the elimination of diseases and the eradication of HCV infection (Table 1).

- the control of liver disease,
- the elimination of liver disease,
- the elimination of HCV infection,
- the eradication of HCV infection.

The first three steps require continued measures to reduce and prevent the HCV transmission, and once elimination and eradication are achieved, intervention measures can be discontinued [9].

The HCV eradication can be achieved only if synergistic prevention and treatment activities are implemented.

There are models of prevention such as the control of blood donors and the prevention of transmission of HCV among persons who inject drugs and models of treatment that involve the elimination of HCV through test and treat strategies that use direct acting antivirals (DAAs) with high efficacy and excellent safety.

The global elimination of HCV before 2030 can be achieved if global and national health organizations build proper models and effective strategies [10,11]:

- screening strategies must be simple and equally effective in high-risk groups and in the general population.
- fast and non-invasive methods for appropriate diagnosis of liver disease are needed.
- strategies for DAA selection must be cost-effective.
- strategy of linkage to care in populations at risk and in marginalized social classes must be specifically designed and applied.
- strategies for obtaining an effective vaccine against HCV infection have yet to be developed.

## 2. Screening Strategies

People with HCV infection remain undiagnosed until they develop symptoms of advanced liver disease or experience abnormal liver tests at clinical check-up. For this reason, new strategies for the diagnosis of HCV infection are needed [12,13]. Screening strategies vary in Europe and the USA depending on the local epidemiology and economic resources. In England, HCV screening is offered to people who inject drugs (PWID) because they represent 80% of infected people, while in France, the national guidelines recommended a screening strategy of 18–59 year old men and pregnant women. Other European countries have chosen screening for vulnerable groups including PWID and prisoners. The United States the Centers for Disease Control and Prevention (CDC) recommends HCV screening for all adults born between 1945 and 1965 because this cohort includes “baby boomers,” people who received blood transfusions or had risk factors before the introduction of blood tests for HCV infection. WHO provided recommendations for risk-based screening, the general population, and birth cohort screening [13]. The first strategy focuses screening on populations with high-risk behavior for HCV infection [14]. The second strategy suggests the screening in populations with a ≥0.1% HCV antibody seroprevalence associated with linkage to care for infected people [13]. The third strategy includes specific birth cohorts of people with known risk of infection [15]. The screening of high-risk groups and cohorts with a high HCV prevalence can result in the diagnosis of a large number of HCV-infected individuals who can be treated, thus increasing the probability of HCV infection control and eradication [16,17].

## 3. Diagnosis Strategies

The screening strategies are aimed primarily at people who have no symptoms of advanced liver disease and have little knowledge of the transmission routes of HCV infection. This strategy must be supported by a health model that provides the diagnosis of HCV infection and disease with fast virological tests and non-invasive methods to individuate the early stages of liver disease. Healthcare organizations that identify HCV infected people must use sensitive and specific tests and access to tests must be easy and fast. Some countries use the Point of Care (POC) for HCV screening. Patients with a positive HCV antibody undertake a quantitative serum HCV RNA test to determine whether they have active HCV infection. EASL and AASLD/IDSA guidelines recommended serum HCV-RNA quantification by sensitive assay with the lowest detection of ≤15 IU/mL [18,19]. The HCV core antigen test (HCV cAg) targets the HCV nucleocapsid peptides 22 (p22) and it is a surrogate marker of viral replication, with very high specificity (>98) [20].

To further simplify the diagnosis pathway, the ideal future algorithm should have the availability of tests to apply directly in the POC or use one test for both screening and diagnosing people with HCV infection.

Rapid diagnostic tests provide results in the same day and could reduce the number of people who are lost to follow-up. Use of oral fluids for the detection of anti-HCV antibodies is currently available and the OraQuick^®^ HCV Rapid Antibody Test has a sensitivity and specificity greater than 99%. The clinical performance of oral tests is equivalent to current laboratory-based EIA. GeneXpert-HCV Viral Load is suitable for detection of serum HCV-RNA in the outpatients and provides results comparable to the laboratory-based test. The test has excellent performance, and its use could be useful in high-risk groups and cohorts where simplicity of care is necessary to implement HCV eradication programs [21]. The POC is an opportunity to plan screening strategies outside healthcare structures or where laboratory-based testing services are not available (e.g., prison services, prevention, and clinical services for PWID).

## 4. Antiviral Treatment Strategies

The main objective of antiviral treatment with direct acting antivirals (DAA) is the sustained virological response (SVR) defined as undetectable HCV RNA 12 weeks after the end of antiviral treatment [18,19]. Several observational studies have shown that SVR reduces liver-related morbidity and mortality and improves quality of life [22]. Therapy is short, well tolerated, and even patients with decompensated cirrhosis or significant comorbidities can be treated. The risk of drug–drug interactions (DDI) is very rare and the minority of patients who developed resistance-associated substitutes (RAS) can be treated with second-line antiviral therapy.

### 4.1. Standard of Care

Antiviral regimens usually consist of at least two different drug classes with different antiviral action and all recommended regimens achieving SVR rates ranging from 90% to 96% depending on the stage of liver disease and viral genotype (GT) [18,19].

Treatment (Table 2) with glecaprevir/pibrentasvir can last 8 weeks in non-cirrhotic and cirrhotic treatment-naive patients, 12 weeks for patients with liver cirrhosis, and 16 weeks for GT 3 patients with liver cirrhosis and/or prior treatment failure. Sofosbuvir/velpatasvir should be administered for 12 weeks independently of staging of liver fibrosis [18,19]. Treatment with grazoprevir/elbasvir is limited in patients with GT 1 or GT4 infection and the duration of treatment varies from 12 to 16 weeks depending on GT, liver fibrosis, and HCV-RNA levels [18,19]. Since the first availability of the DAAs, treatment decisions were based on viral genotype, but today pangenotypic regimens such as sofosbuvir/velpatasvir and glecaprevir/pibrentasvir have high antiviral efficacy against all HCV genotypes. With these assumptions, the fast and easy access to antiviral treatment is a worldwide priority and the therapeutic regimes with pangenotypic drugs can be used in all patients with chronic hepatitis or compensated cirrhosis.

This strategy can be applied in countries where the genotype determination is not available or not affordable and in patient groups in which genotype determination limits the access to antiviral treatment. The simplified treatment without testing for HCV genotypes could facilitate the cascade of care. EASL guidelines [18] recommend that populations who are more difficult to include in the treatment of HCV infection (PWIDs, prisoners, homeless, migrants, people living in rural communities, patients with mental health or substance use disorders, men who have sex with men, sex workers, or indigenous populations) are those who will benefit more from a fast and easy care pathway. The stage of liver fibrosis must be evaluated prior to therapy with simple non-invasive methods, such as FIB-4 or APRI score or Fibroscan, to determine the duration of treatment (8 or 12 weeks) and the indication to surveillance for HCC after the SVR. For patients with an undefined stage of liver fibrosis, a treatment of 12 weeks is recommended regardless of the regimen used.

### 4.2. Special Groups of Patients

#### 4.2.1. Patients with Prior DAA Treatment Failure

After failure of DAA, the development of RAS linked to NS3, NS5A, and NS5B genes is possible. RAS linked to the NS5A gene and NS3 gene can persist for shorter or longer time periods, while RAS related to NS5B are usually rapidly suppressed after treatment cessation due to a significantly impaired viral fitness. Only the combination of sofosbuvir, velpatasvir, and voxilaprevir is approved for the retreatment of patients with prior DAA failure [18] and recommended treatment duration is 12 weeks. Real-world data confirmed that SVR rates >95% can be achieved in pretreated patients independently from the initial treatment regimen [23].

#### 4.2.2. Patients with Impaired Renal Function

Renal excretion is the main elimination pathway of sofosbuvir and its metabolites. Real-world studies show high efficacy of sofosbuvir treatment for patients with severe kidney dysfunction, but these patients also experience higher rates of anemia and worsening of kidney dysfunction [24]. The pangenotypic combination of glecaprevir/pibrentasvir is highly effective and safe in GT2 and GT3 patients with renal impairment and hemodialysis [25]. Alternatively, grazoprevir/elbasvir can be safely administered in patients with GT 1 or 4 infection and severe renal dysfunction [26].

#### 4.2.3. Patients with Decompensated Cirrhosis

Treatment of patients with decompensated cirrhosis is limited to regimens containing sofosbuvir and NS5A inhibitors, while protease inhibitors are not recommended due to the hepatic metabolization. For this reason, grazoprevir/elbasvir, glecaprevir/pibrentasvir, and sofosbuvir/velpatasvir/voxilaprevir combinations cannot be administered. The combination of sofosbuvir and velpatasvir is recommended for those patients, with ribavirin for 12 weeks or without ribavirin for 24 weeks. The rate of SVR of sofosbuvir/ledipasvir plus RBV is 87–96% in patients with Child–Pugh B cirrhosis and 72–85% in patients with Child–Pugh C cirrhosis [18,19].

#### 4.2.4. Patients with Virus Co-Infection

In patients with Hepatitis B Virus (HBV) co-infection, serum HBV DNA levels are usually low, whereas serum HCV-RNA is detectable, and HCV infection is the cause of liver inflammation. Before antiviral treatment of HCV, the replicative status of HBV, including hepatitis D infection, must be evaluated and DAA treatment against HCV should be started with the same recommendations for mono-infected patients [18].

The success of DAA treatment is not impaired by HBV co-infection or HBV treatment. Some studies report an increase in serum HBV DNA levels or HBV reactivation during DAA treatment but so far, the risk of clinical deterioration of liver disease is unpredictable [27]. Current guidelines recommend the concomitant antiviral HBV treatment in HBsAg positive patients with cirrhosis [18,19]. Treatment recommendations for patients with HCV/HIV co-infection do not differ from HCV mono-infected patients. Potential drug interaction between antiretroviral drugs and DAA must be evaluated before choosing a DAA regimen, especially if NS3 protease inhibitors are included in the regimen [19].

#### 4.2.5. Patients with Liver Transplantation

The reinfection after liver transplantation leads to fast development of liver fibrosis and antiviral treatment preserves liver function and increases transplant survival. Transplanted patients showed similar SVR rates compared to non-transplanted patients with the treatment of sofosbuvir/velpatasvir [28]. Potential drug interaction between DAA-containing protease inhibitors and immunosuppressive drugs require special attention, and during DAA treatment serum levels of immunosuppressive drugs need to be closely monitored [29].

## 5. Linkage to Care and Treatment Access

After the diagnosis of HCV infection, all patients should be linked to clinical centers able to provide the liver disease staging and HCV treatment. 

There is a significant number of barriers between diagnosis and access to antiviral treatment. The slowdown in the cascade of care is more pronounced in at-risk groups such as PWIDs or prisoners. PWIDs often have social stigma and difficulty accessing HCV therapy. In a study performed in a suburban area of New Jersey, 237 of 861 PWIDs observed had a positive HCV antibody on screening, but only 16 (6.8%) patients attended the clinical visit, and only 3 (1.3%) received DAA therapy [30].

Use of social and educational support and information services served to enhance patients’ experience and engagement in programs of linkage to care. Several studies clearly demonstrate that identification and designation of resources to facilitate the linkage to care can improve the rate of assessment of treatment and SVR.

DAAs require minimal monitoring and can be delivered effectively by non-specialists and other providers with comparable efficacy [31]. HCV treatment in difficult settings such as substance abuse subjects can overcome access barriers [32].

The use of telehealth technologies can also offer solutions for linkage to care of people residing far from care centers [33]. Telehealth programs range from educational and consultative resources to possible contact with treatment center specialists, including video or telephone calls and online platforms [34].

Access to therapy for all people with HCV infection is essential to improve the outcomes of patients with severe disease and reduce the risk of transmission of the infection in the general population. Access to therapy only for patients with severe disease or for patients in at-risk groups who discontinued substance or alcohol abuse was due to the high cost of drugs.

The high cost of drugs has determined the differences in access to DAA therapy between developed and developing countries and is one of the main barriers to achieving the global eradication of HCV before 2030 as stated by WHO.

The cost reduction and the simplification of access to treatment allow the implementation of eradication programs for groups at risk or for countries with a high prevalence of infection [35,36]. Generic HCV therapies are now available in more than 100 countries with very low costs (less than USD 200) and simple care models were developed to treat the greatest number of people [37].

Multidisciplinary models and strategy programs including general practitioners (GP), specialty pharmacies, nurses, and social workers must be implemented to overcome barriers and improve patients’ access to treatment [38,39].

## 6. Treatment Monitoring and Post-Treatment Follow-Up

Despite the excellent safety and efficacy of HCV therapies, on-treatment monitoring remains an important tool of the care program. Treatment monitoring includes the control of the adherence and therapy completion, identification of drug–drug interactions, management of adverse effects, and assessment of sustained virological response [18,19]. The center that plans access to therapy must engage the patient with a program that includes the duration of therapy, drug delivery, the laboratory requirements, the control of adverse events, and the time to evaluate SVR. With the availability of pangenotypic antiviral regimens with high efficacy and safety, the treatment program could be simplified, which includes a virological control at the beginning and a virological control to evaluate the SVR 12 weeks after the end of the treatment. Pharmacists or non-specialist providers may also contribute directly to therapy monitoring either in a clinic or using telehealth systems [40]. Studies have demonstrated the improved adherence and better prevention of drug–drug interactions and medication errors with the involvement of support staff [41,42]. All patients who complete DAA therapy should be evaluated for SVR at least 12 weeks after completing therapy to confirm the efficacy of therapy and to determine the plan for subsequent clinical follow-up [18,19]. For all patients who achieve SVR, the need for clinical follow-up is scheduled by the degree of liver fibrosis. Patients with mild or moderate liver fibrosis do not require further clinical follow-up. Patients with cirrhosis require hepatocellular carcinoma surveillance and periodic evaluation of portal hypertension [18,19]. Patients who achieve SVR should be informed about the risks of HCV reinfection and reinfection risk varies across cohorts [43,44]. High-risk groups, such as PWID, men who have sex with men, and those with HIV/HCV coinfection, should receive specific education including risk reduction strategies.

## 7. Development of a HCV Vaccine

Development of a vaccine for HCV has been a global goal since the first observations that demonstrated that a significant number of individuals spontaneously clear HCV by means of an appropriate immune response. Several studies have shown that a strong immune response is required to prevent persistence of HCV. In recent years, different forms of preventive and therapeutic vaccines against the HCV have been studied and developed using the techniques of peptide vaccines, recombinant protein vaccines, HCV-like particle, DNA vaccines, and viral vectors expressing HCV genes [45].

Vaccine studies are based on two general principles. First, the spontaneous resolution of HCV infection can protect against persistence of infection after the re-exposure to the virus and the rate of persistence is much lower in second HCV infections compared with that in the first HCV infections, even when the re-infection occurs years later [46].

Second, rechallenge of immune chimpanzees with HCV results in viremia, but with shorter duration and lower peak magnitude than in primary infections.

Prospective studies in PWID showed that 80% of primary HCV infections persisted, while only 20% of secondary infections persisted in subjects who cleared the first infection [47,48]. During infection, a strong and persistent HCV-specific T-cell response is observed in subjects who achieved a spontaneous clearance [49,50]. B cells and rapid induction of cross-reactive neutralizing antibody responses play an active role in the spontaneous recovery of HCV infection [51,52].

HCV mutates with a frequency of nearly one nucleotide per replication cycle and this event is due to the lack of proofreading activity of NS5B RNA dependent polymerase. [53] These frequent mutations, in combination with a short viral half-life and rapid turnover, lead to a high genetic variability which would cause the presence of distinct but closely related HCV variants, known as quasispecies. It is estimated that HCV is 10 times more variable than the human immunodeficiency virus (HIV), and it poses a significant challenge for successful vaccine development [54].

The targets of HCV-specific neutralizing antibodies are the glycoproteins E1 and E2. Recent evidence on HCV glycoprotein structure and their epitopes suggested the rational structure-based antigen design to target specific antibodies. The construction of a vaccine capable of inducing sterilizing immunity will be a difficult challenge, but a vaccine that prevents chronic hepatitis C infections could be a realistic goal in the short term and could have a considerable health impact [55].

With the current availability of highly effective DAAs, it is difficult to propose clinical trials of vaccines in healthy volunteers and cohorts at high risk of infection. Collaborative efforts for the establishment of cohorts in which vaccine clinical trials are conducted are essential, although the ethical implications will have to be considered carefully [56].

## 8. Conclusions

WHO and national health organizations have an ambitious project to eradicate HCV infection in the next 10 years. This project is very difficult to implement, and the SARS-2 pandemic will surely slow down actions to achieve this result. Given the lack of an effective vaccine available to immunize people at risk of developing HCV infection, health organizations will focus on the possibility of treating most people with chronic HCV liver disease. The antiviral drugs available today have high efficacy and excellent safety and can be used in all patients with clinically evident chronic liver disease and in groups that demonstrate risk behaviors to reduce the spread of infection. An information campaign and the implementation of effective linkage to care projects can increase the number of people who will achieve eradication of the infection. To obtain the best results, the virological tests used to diagnose the infection must be easy to use, must have low costs, and must be widespread. The cost of drugs has yet to fall and pangenotypic regimens must be used. Diagnosis of the stage of liver disease must be fast and must use non-invasive methods. Specialist centers must be used to treat the greatest number of patients, but peripheral medical centers must also be used to treat patients who demonstrate risk behaviors or who live far from specialized centers. Patients receiving antiviral therapies should be informed about the efficacy and safety of the therapy and after the end of treatment they should be informed about the need for clinical monitoring and the risk of reinfection.

## Figures and Tables

**Table 1:** Definition of control, elimination, and eradication of viral infection by WHO.

**Control of Disease**
- Reduction in disease incidence, prevalence, morbidity, or mortality to a locally acceptable level as a result of deliberate effort. - Continued intervention measures are required to maintain the reduction.
**Elimination of Disease**
- Reduction to zero of incidence of a specified disease in a defined geographic area as a result of deliberate effort. - Continued intervention measures are required to maintain the reduction.
**Elimination of Infection**
- Reduction to zero of incidence of infection caused by a specific agent in a defined geographic area as a result of deliberate effort. - Continued intervention measures to prevent reestablishment of transmission are required.
**Eradication of Infection**
- Permanent reduction to zero of the worldwide incidence of infection caused by a specific agent as result of deliberate efforts. - Intervention measures are no longer required.

**Table 2:** Standard of care of patients with HCV infection *.

Genotypes	Stage of Liver Disease	Prior Treatment Experience	Sofosbuvir/Velpatasvir	Glecaprevir/Pibrentasvir	Grazoprevir/Elbasvir
Genotypes 1a, 1b, 2, 4, 5 and 6	Chronic hepatitis	Treatment-naive	12 weeks	8 weeks	12 weeks (GT 1b only)
		Treatment-experienced			
	Compensated cirrhosis	Treatment-naive			
		Treatment-experienced		12 weeks	
Genotype 3	Chronic hepatitis	Treatment-naive	12 weeks	8 weeks	NO
		Treatment-experienced		12 weeks	NO
	Compensated cirrhosis	Treatment-naive	12 weeks with Ribavirin	8 weeks	NO
		Treatment-experienced		16 weeks	NO

* adapted by EASL recommendations [18].

## Data Availability

Not Applicable.
